# Prognostic Value of Pretreatment ^18^F-FDG-PET/CT Metabolic Parameters in Advanced High-Grade Serous Ovarian Cancer †

**DOI:** 10.3390/cancers17040698

**Published:** 2025-02-19

**Authors:** Daniela Travaglio Morales, Mónica Coronado Poggio, Carlos Huerga Cabrerizo, Itsaso Losantos García, Cristina Escabias del Pozo, Carmen Lancha Hernández, Sonia Rodado Marina, Luis Domínguez Gadea

**Affiliations:** 1 Nuclear Medicine Department, La Paz University Hospital, 28046 Madrid, Spain; 2 Doctoral School, Universidad Autónoma of Madrid, 28049 Madrid, Spain; 3 Nuclear Medicine Department, Leipzig University Hospital, 04103 Leipzig, Germany; 4 La Paz University Hospital, 28046 Madrid, Spain; 5 Biostatistics Department, La Paz University Hospital, 28046 Madrid, Spain

**Keywords:** PET/CT, ovarian cancer, metabolic parameters, prognosis

## Abstract

Aim: To assess the prognostic value of pretreatment ^18^F-FDG-PET/CT quantitative metabolic parameters in patients with advanced high-grade serous ovarian cancer (HGSOC). Methods: A review of 47 patients diagnosed with advanced HGSOC between 2012 and 2020 in our center was performed, evaluating pretreatment ^18^F-FDG-PET/CT metabolic parameters: maximum standardized uptake value (SUVmax), total lesion glycolysis (TLG) and metabolic tumoral volume (MTV). Two experienced nuclear medicine physicians evaluated the images, thereby obtaining quantitative parameters semiautomatically classifying the volume of interest (VOI) as the target (t): VOI with the highest SUVmax normalized by lean body mass (SUVmax(lbm)), non target (nt) and total (sum of target and non-target VOIs). The disease-free survival (DFS) and overall survival (OS) were calculated. Optimal cutoff values with ROC curves/median values were used. The Correlation between metabolic parameters and DFS/OS was determined using univariate and survival-curves analysis. Results: The median DFS was 18 months (2.5–55) and the OS 33.6 months (2.5–92). The MTVtotal, MTV(t), TLGtotal and TLG(t) were significantly associated with DFS (*p* = 0.005, 0.01, 0.04 and 0.04, respectively). The patients with MTVtotal > 427.8 cm^3^ and MTVtarget > 434 cm^3^ had shorter DFS than the patients with lower values (18.8 versus 31 months and 15.6 versus 30, *p* = 0.02 and 0.01, respectively). The patients with higher TLGtotal and TLG(t) values tended to have worse DFS (*p* = 0.26 and 0.31, respectively). In a multivariate analysis, the MTVtotal was statistically significantly associated with DFS (*p* = 0.003). No correlation was found with OS. Conclusions: Pretreatment MTVtotal and MTV(t) appear to be predictive of relapse in patients with advanced HGSOC.

## Simple Summary

Patients with advanced high-grade serous ovarian cancer (HGSOC) have high relapse and mortality rates. The aim of our work was to assess the prognostic value of pretreatment ^18^F-FDG-PET/CT quantitative metabolic parameters in these patients. Our results show that pretreatment PET metabolic parameters can identify risk groups in advanced high-grade serous ovarian cancer. High metabolic tumor volume (MTV) and total lesion glycolysis (TLG) are associated with shorter disease-free survival (DFS), with MTV being the strongest predictor. Pretreatment MTV may be able to predict high risk of relapse in patients with advanced HGSOC at initial staging.

## 1. Introduction

Ovarian cancer (OC) is the third main cause of death related to gynecologic cancer, and it is the fourth most commonly diagnosed gynecologic cancer in the world [1].

Epithelial ovarian cancer is the most common histologic type of OC, and high-grade serous ovarian carcinoma (HGSOC) represents 70 to 80% of this histologic sub-type. Clinically, HGSOC has an aggressive pattern, and most cases of HGSOC are diagnosed in advanced stages. Generally, the disease spreads intraperitoneally, resulting in a lack of symptoms in early stages [2]. The prognosis of advanced HGSOC is poor. Fortner et al., from a Norwegian cohort of 3664 cases of OC between 2012 and 2021, found that 83.8% of deaths corresponded to patients with HGSOC, most of them being stage III or IV. Five-year relative survival rates of 45.7% and 25.9% were reported for HGSOC in stage III and IV, respectively [3]. Furthermore, almost 80% of women with OC at an advanced stage who respond to first-line chemotherapy relapse within a median time period of 18 months [4]. Other authors reported disease-free survival (DFS) of 21.9 and 13.8 months, with and without BRCA mutation, respectively, in a population of OC at an advanced stage [5]. Therefore, it is necessary to identify patients at high risk of relapse and to investigate different maintenance treatments to reduce recurrence and determine follow-up.

Positron emission tomography/computed tomography with ^18^F-Fluorodeoxyglucose (^18^F-FDG- PET/CT) is a widely applied molecular imaging technique in the oncological field for cancer diagnosis, staging, recurrence detection and response evaluation [6]. ^18^F-FDG uptake is proportional to glucose metabolism, which is increased in many tumors. Usually, ^18^F-FDG uptake is measured semi-quantitatively, through a standardized uptake value (SUV). The maximum uptake value (SUVmax) represents the highest point of metabolism within the tumor [7]. Some studies have shown that SUVmax does not reflect the entire tumoral nature and, further, that it is sensitive to image noise [8]. Image noise can limit reproducibility and can induce positive bias. Statistical noise may alter the pixel values of a uniform region of tracer accumulation. As SUVmax considers the highest pixel value, there is an overestimation of the real value [9]. There are volume-based metabolic parameters, such as metabolic tumor volume (MTV) and total lesion glycolysis (TLG), which can reflect whole-tumor characteristics.

Recent studies have shown that MTV and TLG are significant prognostic factors in many types of cancer [10,11]. In ovarian cancer, there is evidence in the literature suggesting the prognostic value of ^18^F-FDG-PET/CT metabolic parameters [12,13,14]. Many studies have investigated the prognostic value of those parameters after initial treatment [13,15]. So far, only a few publications have investigated the utility of metabolic parameters before initial treatment in patients already diagnosed with ovarian carcinoma, and they demonstrate that the calculation of tumor burden at this point is useful to predict recurrence and survival, although they have some limitations, such as different stages and tumor histologies [16,17,18,19,20,21]. Therefore, more data are needed to support the pretreatment prognostic value of ^18^F-FDG-PET/CT metabolic parameters in a cohort of patients with the same histology and stage, such as advanced HGSOC.

## 2. Materials and Methods

### 2.1. Patients

We retrospectively reviewed patients diagnosed with and treated for advanced HGSOC (FIGO staging III and IV) in our institution, who underwent PET/CT imaging prior to initial therapy between January 2012 and September 2020, followed by surgery or chemotherapy. Initially, all consecutive PET/CT studies from patients with OC were selected. Patients were excluded if (1) they did not have HGSOC (the histological diagnosis was made by a dedicated pathologist specializing in gynecological tumors, member of the Multidisciplinary Gynecological Tumor Board of La Paz University Hospital), (2) they underwent PET/CT for diagnosis of relapse or treatment monitoring for OC, (3) they had initial FIGO stages (I–II), (4) they had received any kind of treatment before PET/CT, or (5) they had undergone adnexectomy for biopsy before PET/CT. The staging and treatment decisions for our sample were made by the tumor board. Age, date of diagnosis, histological type of tumor, FIGO staging, treatment data, PET/CT date, date of relapse, actual status and date of death/last consultation were collected. All the patients were treated and followed up, clinically and radiologically, according to the tumor board. Clinical data were extracted from Dedalus HCIS Healthcare Information System. This study was approved by the ethics committee of our institution.

### 2.2. PET/CT Imaging Technique

**Figure 1:**
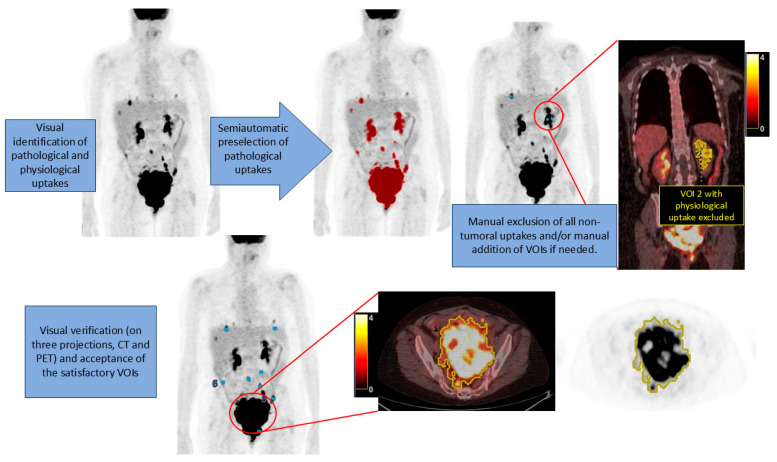
Reading and segmentation method for PET/CT images. VOI: volume of interest. Blue dots: locations of the VOI. Yellow line: VOI contour. Color bar indicates SUVmax values.

All patients fasted for at least 4 h prior to the PET/CT acquisition. The images were performed 60 min after intravenous injection of a standard dose of ^18^F-FDG. Patients lay supine with the arms above the head. A low-dose CT without iv contrast was acquired from the skull base to the proximal thighs, followed by 2D or 3D PET acquisition (2–4 min per bed, depending on the scanner). The images were reconstructed using ordered subset expectation maximization with CT attenuation correction. The studies were acquired in all the PET/CT scanners concerted by our institution. Scanner, acquisition and reconstruction parameters are shown in Supplementary File S1. A visual interpretation was performed by two nuclear medicine physicians with wide experience in PET and CT, respectively. The readers were only aware that the patients met the inclusion criteria. Subsequently, they performed a semiautomatic segmentation for every patient, using the PET VCAR application from the AW VolumeShare 7 version AW4.7 in the Advantage Workstation (GE Healthcare Medical Systems, San Francisco, CA, USA).The visual analysis of metabolic and morphological data was realized in the Single Review PET/CT workflow, through which pathological findings in relation to OC were identified. Subsequently, the physicians realized semiquantitative analysis using the PET/CT VCAR workflow, with the following steps: they fixed a SUVmax threshold with which the software highlighted all pathologic PET uptakes meeting PET Response Criteria in Solid Tumors (PERCIST), denoted as “pre-selected uptakes”. The SUVmax was normalized for lean body mass (SUVmax(lbm)), which was different from body weight normalization, since normalization based on lean body mass instead of weight alone is recommended for better precision and intercompatibility [22]. Next, segmentation was performed, drawing a volume of interest (VOI) semiautomatically around the pre-selected uptakes in the transaxial, sagittal and coronal projections. The VOIs were made using boundaries of voxels with a threshold of 41% of SUVmax of the lesion to define the tumor volumes. The VOIs enclosed the primary tumor and metastatic lesions of HGSOC. The quality of the delineation was visually confirmed. Adequate VOIs were accepted. Inadequate VOIs, including physiological uptake in organs such as heart, liver, kidneys, ureters and bladder, in addition to inflammatory lesions or other non-tumoral or primary neoplasms, were excluded manually from the VOIs. In the segmentation process, lesions smaller than 1 cm^3^ were not included unless they had a considerable uptake, to prevent uptake underestimation from the partial volume error [8]. Moreover, lesions were not added manually, except for the most obvious ones. Subsequently, metabolic parameters were measured. MTV and TLG were calculated. MTV was measured as the functional volume (cm^3^) of the segmented lesions. TLG was calculated as (MTV) × (SUVmean(lbm)). The software classified the contoured lesions as target or non-target, identifying a target VOI (with maximum SUVmax(lbm)) for each patient independently of the location. The non-target parameters were the result of the sum of parameters of all non-target VOIs of each patient. Finally, we calculated the sum of parameters (target and non-target) to obtain the total value for each patient. Figure 1 exemplifies the reading and segmentation of PET/CT.

### 2.3. Clinical End Points and Follow-Up

Data of relapse, death and other clinical information were collected. Relapse was diagnosed based on clinical suspicion due to worsening of symptoms and increased CA-125 levels and confirmed by imaging studies. Time to relapse was defined as time between date of diagnosis and date of relapse/progress. Time to death was defined as the time between date of diagnosis and death related to primary OC.

### 2.4. Statistical Analysis

The description of quantitative data was performed through mean or median values, depending on the distribution. Since our sample size was close to 50 patients, the normality of the variables was studied through Kolmogorov–Smirnov test. A replication with Shapiro–Wilk test established the same distributions.

Metabolic parameters were compared with FIGO staging using the Mann–Whitney analysis. Kaplan–Meier analysis was made to obtain DFS and overall survival (OS) values. Metabolic parameters (SUVmax, MTV and TLG, target or non-target) were correlated with DFS and OS using a Cox proportional hazard regression test with 95% confidence interval. Before this, the Ph hazard assumption was calculated and supported using the cox.zph() function of R software version 4.0.2.

Optimal cutoff values for MTV and TLG were searched through receiver operating characteristic analysis (ROC). The median value was chosen as cutoff in those parameters without an acceptable discriminating threshold in ROC curves.

Survival analysis was performed with Log Rank (Mantel–Cox) test categorizing groups according to cutoff values, with posterior analysis of risk through Cox regression test (Supplementary File S3). A multivariate analysis of the imaging and clinical findings was performed.

A *p* value of ≤0.05 was considered statistically significant. The statistical analysis was made using SAS 9.3 (SAS Institute, Cary, NC, USA).

## 3. Results

### 3.1. Patient Characteristics

**Figure 2:**
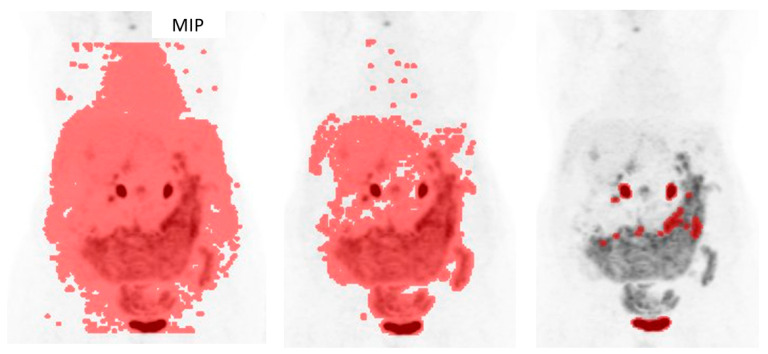
Example of exclusion criteria: Low background–tumor ratio. The software could not correctly highlight and preselect the tumoral lesions: either a fair amount of physiological uptake was included, or most of the tumor was excluded. This would have forced manual segmentation. MIP: maximum intensity projection. Red indicates the preselected volumes of interest (VOIs).

**Figure 3:**
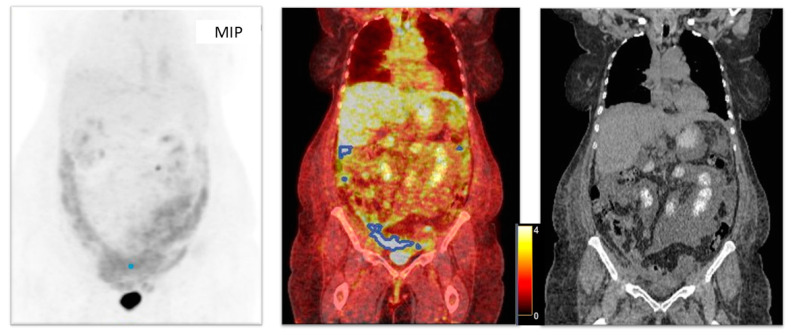
Example of exclusion criteria: non-target volume 0. Only one VOI was semiautomatically drawn (blue dot and lines) and categorized as target. Thus, the non-target volume was zero. The patient was excluded, since it was not possible to apply our methodology. MIP: maximum intensity projection. Blue line indicates the volume of interest (VOI). Blue dot: locations of the VOIs. Color bar indicates SUVmax values.

Initially, studies of 260 consecutive patients with OC were obtained for the period between January 2012 and September 2020. Twenty-six patients were excluded as they had histologies differing from HGSOC. In total, 137 patients were eliminated because PET/CT was performed for treatment monitoring or relapse diagnosis. Seven patients were excluded since they had initial FIGO stages (I–II). In total, 24 patients were removed as they underwent an exploratory laparoscopy with some cytoreduction or received some cycle of chemotherapy before PET/CT acquisition, and four patients had an adnexectomy as a biopsy before PET/CT as well. Finally, 62 patients with HGSOC, advanced stage (III–IV FIGO staging) and basal PET/CT were collected. Six patients had no follow-up. Images were not available for analysis for three patients. Subsequently, 53 patients were candidates for segmentation. Because of low tumor-to-background uptake ratios and technical issues, metabolic parameters could not be calculated for four and two patients, respectively (Figure 2 and Figure 3). The technical issue consisted in the impossibility of calculating target/non target volumes, since the result of the semiautomatic segmentation was only one VOI; thus, we could not calculate a non-target volume, and it was not possible to apply our methodology.

**Figure 4:**
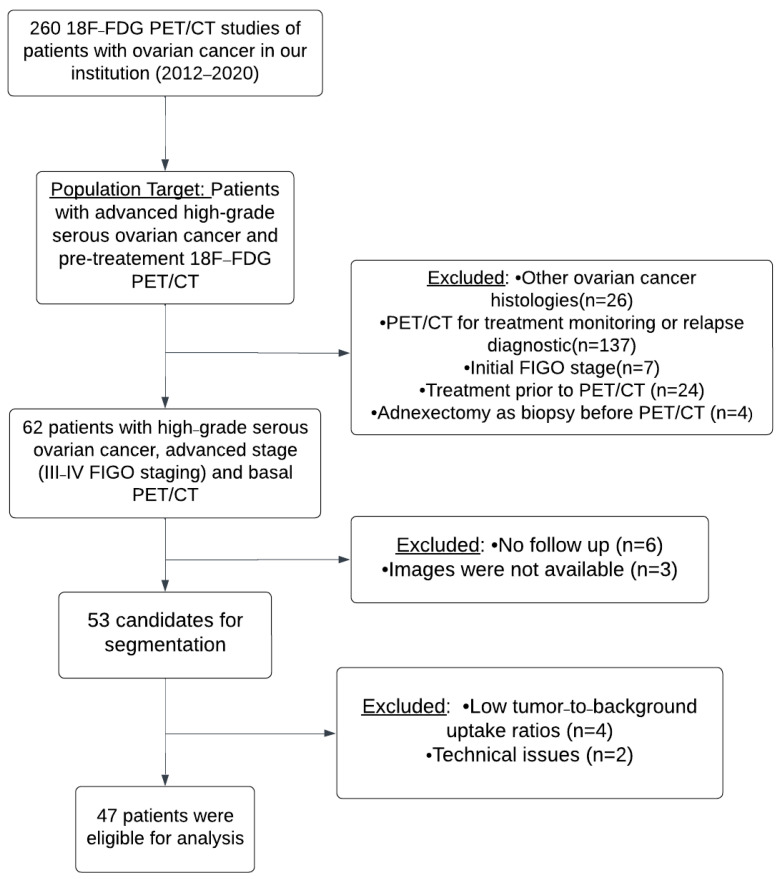
Flowchart showing the patient selection process.

Finally, 47 patients were eligible for analysis, with a mean age of 61 years (range 33–84), all of them Caucasian (Figure 4 and Table 1). The mean follow-up was 33.7 months (20.7–46). In total, 13 patients were III FIGO stage (27.7%) and 34 patients were stage IV (72.3%). Our cohort of patients were treated with neoadjuvant chemotherapy based on carboplatin–paclitaxel, as well as interval debulking surgery in 25 patients (53.2%), primary cytoreductive surgery followed by adjuvant chemotherapy in 12 patients (25.5%). Chemotherapy alone was used in 10 patients (21.3%). In total, 33 patients (70.2%) went through complete cytoreductive surgery at some point in the treatment (primary cytoreductive surgery or interval debulking surgery). Relapse and exitus occurred in 70.2% and 39% of the patients, respectively. The mean values for DFS and OS were 18 and 33.6 months, respectively (Table 1).

### 3.2. Metabolic Parameters and FIGO Stage (III–IV)

We did not find any statistically significant associations between metabolic parameters and FIGO stage.

### 3.3. Metabolic Parameters and DFS

A statistically significant correlation was found between MTVtotal and MTVtarget and DFS (*p* = 0.005 and *p* = 0.01, respectively). For every 100 units of incremented MTVtotal and MTVtarget, the risk of relapse increased by 7.2% and 6.3%. Similarly, we found that TLGtotal and TLGtarget were significantly correlated with DFS (*p* = 0.04 and *p* = 0.04, respectively). For every 100 units of incremented TLGtotal and TLGtarget, the risk of relapse increased by 1.7% and 1.6%, respectively.

#### 3.3.1. Cutoff Values

The ROC curves from the significative parameters are shown in Figure 2. The AUC for MTVtotal was 0.658, and for MTVtarget, TLGtarget and TLGtotal, the values were 0.643, 0.606 and 0.597, respectively. The optimal cutoff value for MTVtotal was 427.8 cm^3^, with a sensitivity of 78.8% and a specificity of 57.1%. Median values were used to choose the cutoff values for MTVtarget, TLGtotal and TLGtarget (Table 2).

#### 3.3.2. Groups Categorized by DFS

**Figure 5:**
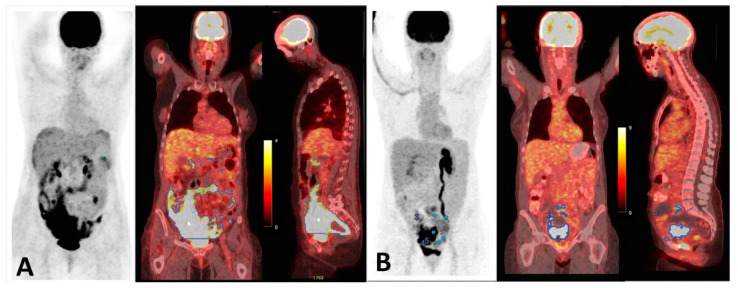
(**A**) A 54-year-old patient with HGSOC. MTVtotal: 1204 cm^3^, MTVtarget: 1186 cm^3^, TLGtarget 3768.6 mL × cm^3^ and TLGtotal: 3777.4 mL × cm^3^. DFS= 4 months. (**B**) A 50-year-old patient with HGSOC. MTVtotal: 194.8 cm^3^, MTVtarget: 152 cm^3^, TLGtarget: 542.6 mL × cm^3^, TLGtotal: 261.2 mL × cm^3^. DFS = 44.9 months.

High values of MTVtotal and MTVtarget were correlated with worse DFS than low values (Figure 3), with hazard ratios (HRs) of 2.6 (*p* = 0.02) and 2.3 (*p*= 0.01), respectively. The mean DFS values were 31 months for patients with MTVtotal < 427.8 cm^3^ versus 18.8 months in patients with MTVtotal > 427.8 cm^3^. In addition, the mean DFS values were 30 months for patients with MTVtarget < 434 cm^3^ compared to 15.6 months in patients with MTVtarget > 434 cm^3^. An example of two different patients with high and low tumor burden is shown in Figure 5.

**Figure 6:**
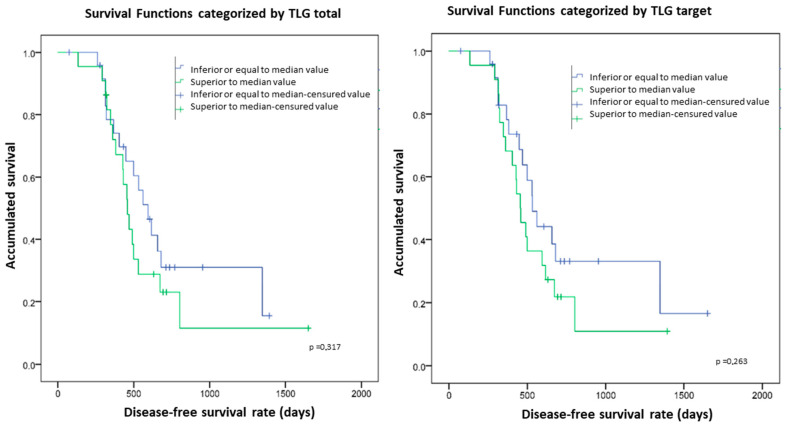
Survival functions categorized by TLGtotal and TLGtarget. The patients with higher values of TLGtotal and TLGtarget tended to exhibit worse DFS than those with lower parameters, although these differences were not statistically significant (*p* = 0.31 and 0.26, respectively).

The patients with median TLGtarget and TLGtotal higher than 1414 cm^3^ and 1818.3 cm^3^, respectively, tended to have worse DFS than those with lower values, although these differences were not statistically significant (*p* = 0.26 and 0.31, respectively) (Figure 6).

A multivariate analysis of the metabolic parameters was performed. It showed that TLGtotal, TLGtarget, VMTtarget and VMTtotal were related to each other, so it was not possible to find a significant independent predictive factor. However, MTVtotal was the strongest predictor (*p* = 1.16). A multivariate analysis of the clinical and metabolic parameters with the more significant variables MTVtotal, stage and age was performed. MTVtotal was statistically significantly associated with DFS (*p* = 0.003).

### 3.4. Metabolic Parameters and OS

We did not find any associations between metabolic parameters and overall survival.

## 4. Discussion

Metabolic analysis has been investigated in many oncologic processes as a prognostic marker at diagnosis [23,24,25]. In OC, some publications have explored functional imaging of metabolic characteristics and its prognostic value with different histologies and stages [13,16,17,18]. The aim of our work was to study the relationship between the metabolic parameters measured in pretreatment ^18^F-FDG-PET/CT and clinical outcomes in patients with advanced HGSOC.

In accordance with other authors [16,17,18], we found that MTV and TLG have a significant correlation with disease recurrence: patients with high tumor burden measured by MTV and TLG have a higher risk of relapse, with MTV being the strongest predictor. This is an obvious finding considering that the patients were at all stages (limited and advanced), but it represents an important contribution in the subgroup of patients with advanced disease.

Liu et al. [20] did not find any correlations between MTV or TLG in primary tumors or omental metastases and DFS. We found that both target and total metabolic volumes were correlated with DFS, even though MTVtotal was found to be the strongest prognostic factor.

The median metabolic volumes in our group are higher than those in other studies because we only included advanced tumors (stages III and IV). The cutoff values for MTV and TLG are also higher in our study, with similar AUC values to those of other authors [16,17,18].

The median SUVmax in our group is high (10.4), in line with other studies [17,20]. However, our results support the argument that volume-based parameters are more accurate as prognostic factors than SUVmax. This is a controversial point in the literature: while some authors demonstrate that higher ^18^F-FDG uptake is associated with more chemosensitivity and better DFS [20], others argue that lower SUVmax in primary tumors correlates with better survival [17,21]. A possible reason for this discordance is the inclusion of different histologic subtypes.

Unlike other authors [16,21], we did not find any associations between FIGO stage (III–IV) and metabolic parameters. This could be due to differences between the stages and histologies of the samples.

In contrast with other authors [20,26], we did not find any associations between metabolic parameters and OS. Possible reasons for this could be the small number of patients in our group, as well the different treatments.

Our work has several limitations. Firstly, it was a retrospective study with a small number of patients and, therefore, it probably had selection bias. To date, all the published data concerning basal PET/CT metabolic parameters in OC are also retrospective and mostly have a small number of patients [16,19,20], but our study includes only one histologic type and only patients in advanced stages. Another drawback is that the PET/CT acquisitions were made in different scanners. Metabolic semiquantitative parameters may be susceptible to changes between scanners. Nonetheless, our results might suggest the strong value of metabolic parameters despite the technical differences.

Furthermore, HGSOC, as a heterogeneous disease, has a variety of treatment options. Our sample was treated differently according to the principles of HGSOC therapy. This could have exerted an impact on the dissimilarity in patient outcomes due to the intrinsic influence of the treatment, which would have limited the value and reliability of our results. For this reason, further prospective studies with a more homogeneous sample are needed.

Low ^18^F-FDG tumor uptake represents an important difficulty in the segmentation process and could be a technical limitation of volume-based parameters compared to SUVmax. The low ^18^F-FDG tumor uptake might be explained by the histological tumoral heterogeneity of HGSOC, including, for example, the variance of GLUT 1 transporter expression [27] or the morphological pattern of spread. These patients had miliary peritoneal carcinomatosis, ascites and pleural effusion, conforming a different morphological and metabolic pattern despite the histologic subtype being the same. We excluded four patients with this characteristic. Other authors have excluded such patients from their analyses as well [17,19]. This also might have led to selection bias, since we only included patients with high tumor uptake.

The most common metastasis site in ovarian cancer is the omentum [28], and disease dissemination through the abdominal cavity is frequently found in HSOC. In this context, segmentation can be really challenging because of the multiple and small lesions located in proximity to organs with physiological FDG uptake. Semiautomatic segmentation requires meticulous selection of pathological areas, differentiating them from physiological uptake to avoid miscalculations. To overcome this difficulty, some authors have tried to find a representative parameter for tumor burden; for example, Liu et al. [20] calculated metabolic volumes both in primary tumors and in omental metastases. Most of the previous studies used different segmentation methods with different delineation thresholds, the most frequent being the semi-automatic method, which is performed by clicking on lesions with a delineation threshold of 40% SUV [16,17,20,21]. We used software with pre-selection of the pathologic lesions, since it is less time-consuming and more standardized. Moreover, we used a threshold of 41% of SUV(lbm). All these variations could have affected the target and non-target VOIs. Nevertheless, the designation of targets by SUVmax(lbm) could have more reproducibility [29]. Although there is some controversy over the best segmentation method for delineating lesions, some authors have demonstrated that there are no significative effects between them to obtain quantitative imaging features [30]. It is important to develop further automatic segmentation methods, with the designation of targets through high uptake being a possibility. Thus, these results should be confirmed by further investigation.

Measurement of tumor volumes can be performed based on morphological imaging (CT/MRI) or PET/CT imaging. The use of ^18^F-FDG PET has limitations in the primary detection of ovarian cancer and small peritoneal tumor volumes [31]. However, some authors reported that it is more accurate than MRI and CT for lymph node detection and more reliable than CT for detection of supradiaphragmatic disease, peritoneal metastases, organ involvement and recurrent disease [31,32,33]. Additionally, PET information is more accurate for the calculation of tumor burden [16]. The use pf ^18^F-FDG PET/MRI seems a promising technique. The combination of both modalities is superior to MRI alone for estimating the total spread of carcinomatosis in OC [34].

## 5. Conclusions

In conclusion, we have studied patients with advanced HGSOC and pretreatment with ^18^F-FDG-PET/CT. In this context, ^18^F-FDG PET/CT is very useful for identifying patients with MTVtotal > 427.8 cm^3^ and MTVtarget > 434 cm^3^ as a subgroup with higher risk of relapse and could contribute to choosing the initial treatment strategy for these patients. Our results should be considered with caution due to the small sample, the single-institution nature of the study and the use of multiple scanners. For these reasons, further investigations on metabolic parameters in a population with advanced HGSOC are needed. Moreover, the variation of the metabolic parameters between pre- and post- treatment ^18^F-FDG-PET/CT, their prognostic value and their impact on the assessment of the primary treatment would be very interesting fields of research for future studies.

## Figures and Tables

**Table 1:** Patients’ characteristics (*n* = 47).

Characteristic	Patients
**Total patients**	47
**Mean age, years (SD)**	61 (11.7)
**FIGO stage**	13 (27.7%)
**III**	34 (72.3%)
**IV**	
**Treatment** Neoadjuvant chemotherapy + interval debulking surgery	25 (53.2%)
Primary cytoreductive surgery + adjuvant chemotherapy	12 (25.5%)
Chemotherapy	10 (21.3%)
**Median follow-up duration, months (Q1–Q3)**	29 (20.7–46)
**Mean (SD)**	33.7 (19.9)
**Median DFS, months (Q1–Q3)**	14.9 (10.7–18.2)
**Mean (SD)**	18 (10.1)
**Median OS, months (Q1–Q3)**	26.3 (12.7–39.2)
**Mean (SD)**	33.6 (20)

SD: standard deviation. Q: quartile.

**Table 2:** Median values of metabolic parameters.

Metabolic Parameter	Median (Quartiles 1 and 3)
SUV(lbm)max	10.4 (8.6–12.9)
SUVmean	4.2 (3.4–6.6)
TLG (cm^3^)	1818.3(1084.9–3777.4)
TLG target (cm^3^)	1414 (403.1–2925.9)
TLG no target (cm^3^)	122.5 (43.1–848.5)
MTV (cm^3^)	584.9 (351.4–1204.4)
MTV target (cm^3^)	434 (121–1044)
MTV no target (cm^3^)	46.7 (14.8–261.3)

SUV(lbm)max: normalized for lean body mass maximum uptake value, MTV: metabolic tumor volume, TLG: total lesion glycolysis.

## Data Availability

The datasets generated and analyzed during the current study are available in the following files, attached to the submission: Patients data.xlsx (Supplementary File S2), Results 1.pdf (Supplementary File S4) and Results 2.pdf (Supplementary File S5).

## Supplementary Materials

The following supporting information can be downloaded at: https://www.mdpi.com/article/10.3390/cancers17040698/s1, File S1: Acquisition and reconstruction parameters + patients; File S2: Patients data; File S3: Multivariate clin + MTV_CLEAN; File S4: Results 1; File S5: Results 2.
