# Supplementary material for: Prognostic Value of Pretreatment 18F-FDG-PET/CT Metabolic Parameters in Advanced High-Grade Serous Ovarian Cancer
Source: Cancers (Basel). 2025 Feb 19;17(4):698. doi: 10.3390/cancers17040698 (PMC11853401; doi:10.3390/cancers17040698)
Supplement: Supplementary file 1 [file cancers-17-00698-s001.zip › File S1. Acquisition and reconstruction parameters + patients.pdf]

SIEMENS Siemens Medical Solution, Knoxiville, TN, USA
GE MEDICAL SYSTEMS General Electric Medical Systems, Milwaukee, USA

**Manufacturer**	GE MEDICAL SYSTEMS	GE MEDICAL SYSTEMS	SIEMENS	GE MEDICAL SYSTEMS	GE MEDICAL SYSTEMS	SIEMENS
**Model**	DISCOVERY - LS	Discovery 690	Biograph 6	Discovery ST	Discovery STE	Biohgraph128Edge
Number of patients	36	2	5	1	1	2
2D/3D Adquisition	2D	3D	2D	2D	3D	3D
Detector elements	BGO (bismuth germanate)	LYSO (lutetium-yttrium-orthosilicate)	LSO (lutetium oxy-orthosilicate)	BGO	BGO	LSO
Frame Duration [s]	240000	120000	162000	240000	180000	90000
Frame Duration [min]	4	2	2.7	4	3	1.5
Reconstruction Diameter [mm]	500	700	682	600	700	726
Field of View Dimensions(s) (transverse/axial) [mm]	550\153	700\153	605/162	700\157	700\153	700/200
Reconstruction Method	OSEM 2D	OSEM 3D+ TOF/PSF	OSEM 2D	OSEM 2D	OSEM 3D	OSEM 3D+ TOF/PSF
dead-time, randoms, scatter, decay Correction	Yes	Yes	Yes	Yes	Yes	Yes
Attenuation Correction Method	Yes (CT-based)	Yes (CT-based)	Yes (CT-based)	Yes (CT-based)	Yes (CT-based)	Yes (CT-based)
Matrix size	128x128	192/192	168x168	128x128	128x128	440x440
Slice Thickness [mm]	4.25	3.27	5.00	3.27	3.27	5.00
Pixel Spacing[mm]	3.906\3.906	3.646\3.646	4.063\4.063	4.688\4.688	5.469\5.469	1.650\1.650

TOF: time- of-fly

PSF: point spread function
OSEM: ordered subset expectation maximisation
