# Supplementary material for: Prognostic Value of Pretreatment 18F-FDG-PET/CT Metabolic Parameters in Advanced High-Grade Serous Ovarian Cancer
Source: Cancers (Basel). 2025 Feb 19;17(4):698. doi: 10.3390/cancers17040698 (PMC11853401; doi:10.3390/cancers17040698)
Supplement: Supplementary file 1 [file cancers-17-00698-s001.zip › File S3. Multivariate clin + MTV_CLEAN.pdf]

# MODEL FOR MTV TOTAL

## Cox regression

**Variables in the equation**

| | | B | SE | Wald | gl | Sig. | Exp(B) | 95.0% CI for Exp(B) | |
|---|---|---|---|---|---|---|---|---|---|
| | | | | | | | | Inferior | Superior |
| Step1 | VMT total | ,001 | ,000 | 6,277 | 1 | ,012 | 1,001 | 1,000 | 1,001 |
| | Age | ,037 | ,018 | 4,199 | 1 | ,040 | 1,037 | 1,002 | 1,074 |
| | Stage_III_IV | 1,015 | ,448 | 5,134 | 1 | ,023 | 2,759 | 1,147 | 6,639 |
| | Type of treatment | ,165 | ,232 | ,504 | 1 | ,478 | 1,179 | ,748 | 1,858 |
| Step2 | VMT total | ,001 | ,000 | 8,933 | 1 | ,003 | 1,001 | 1,000 | 1,001 |
| | Age | ,037 | ,018 | 4,306 | 1 | ,038 | 1,038 | 1,002 | 1,075 |
| | Stage_III_IV | 1,004 | ,447 | 5,047 | 1 | ,025 | 2,729 | 1,137 | 6,554 |

The final model would be the one in step 2 but if you want the one that adjusts for the three clinical variables you would keep the one in step 1.
