# Supplementary material for: Prognostic Value of Pretreatment 18F-FDG-PET/CT Metabolic Parameters in Advanced High-Grade Serous Ovarian Cancer
Source: Cancers (Basel). 2025 Feb 19;17(4):698. doi: 10.3390/cancers17040698 (PMC11853401; doi:10.3390/cancers17040698)
Supplement: Supplementary file 1 [file cancers-17-00698-s001.zip › File S4. Results 1.pdf]

GENERAL DESCRIPTIVE

1) Categorical variables - Frequencies

Relapse

		Frequency	Percentage	Valid percentage	Cumulative percentage
Valid	no	14	29,8	29,8	29,8
	Yes	33	70,2	70,2	100,0
	Total	47	100,0	100,0	

Exitus

		Frequency	Percentage	Valid percentage	Cumulative percentage
Valid	no	28	59,6	60,9	60,9
	Yes	18	38,3	39,1	100,0
	Total	46	97,9	100,0	
Lost	System	1	2,1		
Total		47	100,0		

Current Situation

		Frequency	Percentage	Valid percentage	Cumulative percentage
Valid	0	10	21,3	21,3	21,3
	1	8	17,0	17,0	38,3
	2	12	25,5	25,5	63,8
	3	16	34,0	34,0	97,9
	4	1	2,1	2,1	100,0
	Total	47	100,0	100,0	

2) Continuous variables

Normal Testing

Kolmogorov-Smirnov test for a sample

	N	Normal parameters[a,b]		Maximum extreme differences			Test statistic	Asymptotic sig (bilateral)
		Stocking	Deviation standard	Absolute	Positive	Negative		
Age at diagnosis	47	61,51	11,712	,096	,068	-,096	,096	,200[c,d]
Ca 125 at diagnosis	47	2851,562	4735,7765	,277	,248	-,277	,277	,000[C]
SUVmax	47	12,4706	8,49263	,285	,285	-,207	,285	,000[C]
SUVpeak	47	9,1034	4,63441	,231	,231	-,144	,231	,000[C]
TLG	47	2561,1445	2039,11189	,175	,175	-,119	,175	.001[c]
TLG target	47	2053,8964	2114,48588	,202	,202	-,169	,202	,000[C]
TLG no target	47	505,5185	761,68401	,292	,292	-,255	,292	,000[C]
MTV Target	47	653,9709	728,05304	,214	,214	-,185	,214	,000[C]
MTV No target	47	170,3729	250,81253	,304	,304	-,249	,304	,000[C]
Total MTV	47	852,1410	749,93790	,188	,188	-,148	,188	,000[C]

a. The test distribution is normal.

b. It is calculated from data.

c. Lilliefors significance correction.

d. This is a lower limit of true significance.

Age at diagnosis remains normal (p=0.200) while the rest of the variables do not: SUVmax (p<0.001),

SUVpeak (p<0.001), TLG (p=0.001), TLG target (p<0.001), TLG no target (p<0.001), MTV target (p<0.001), MTV no target (p<0.001) and MTV total (p<0.001).

Mean and standard deviation

Case Summaries

Age at diagnosis

Minimal	Mean	Standard deviation	Maximum	N
33	61,51	11,712	84	47

Median and interquartile range

Statistical

	N		Percentiles		
	Valid	Lost	25	50	75
SUVmax	47	0	8,6500	10,4400	12,9500
SUVpeak	47	0	6,9900	7,9100	9,7900
TLG	47	0	1084,9200	1818,3600	3777,4300
TLG target	47	0	403,1400	1414,0300	2925,9500
TLG no target	47	0	43,1800	122,5300	848,5600
MTV Target	47	0	121,0000	434,0000	1044,0000
MTV No target	47	0	14,8400	46,7700	261,3800
Total MTV	47	0	351,8400	584,9890	1204,4200

ASSOCIATION OF NUCLEAR PARAMETERS WITH DFS

Cox Regression - SUVmax

Variables in the equation

	B	SE	Wald	Gl	Sig.	Exp(B)	95.0% CI for Exp(B)	
							Inferior	Superior
SUVmax	,004	,020	,033	1	,857	1,004	,966	1,043

Covariate means

	Mean
SUVmax	12,635

p=0.857 does not affect DFS

Cox Regression - SUVpeak

Variables in the equation

	B	SE	Wald	Gl	Sig.	Exp(B)	95.0% CI for Exp(B)	
							Inferior	Superior
SUVpeak	,003	,038	,005	1	,941	1,003	,931	1,080

Covariate means

	Mean
SUVpeak	9,225

p=0.941 does not affect DFS

Cox regression - TLG

Variables in the equation

	B	SE	Wald	Gl	Sig.	Exp(B)	95.0% CI for Exp(B)	
							Inferior	Superior
TLG	,000	,000	3,965	1	,046	1,000	1,000	1,000

Covariate means

	Mean
TLG	2587,569

p=0.046 TLG affects DFS
For every unit increase in TLG increases the risk of relapse by 0.02% For every 10 units increase in TLG increases risk of relapse by 0.18%
For every 100 units of increase in TLG increases the risk of relapse by 1.7 9%

Cox regression - TLG target

Variables in the equation

	B	SE	Wald	Gl	Sig.	Exp(B)	95.0% CI for Exp(B)	
							Inferior	Superior
TLG target	,000	,000	4,139	1	,042	1,000	1,000	1,000

Covariate means

	Mean
TLG target	2082,087

p=0.042 TLG target affects DFS
For each unit increase in TLG target, the risk of relapse increases by 0. 02%
For every 10 units of increase in TLG target, the risk of relapse increases 0.17%
For every 100 units of increase in TLG target, the risk of relapse

increases by 1.68%

Cox regression - non-target TLG

Variables in the equation

	B	SE	Wald	Gl	Sig.	Exp(B)	95.0% CI for Exp(B) Inferior	Superior
TLG no target	,000	,000	,131	1	,718	1,000	,999	1,000

Covariate means

	Mean
TLG no target	503,715

p=0.718 does not affect DFS

Cox regression - MTV target

Variables in the equation

	B	SE	Wald	Gl	Sig.	Exp(B)	95.0% CI for Exp(B) Inferior	Superior
MTV Target	,001	,000	6,446	1	,011	1,001	1,000	1,001

Covariate means

	Mean
MTV Target	658,427

p=0.011 MTV target is associated with DFS.
For each unit increase in MTV target, the risk of relapse increases by 0.06%
For every 10 units of increase in MTV target increases the risk of relapse by 0.64%
For every 100 units of increase in MTV target increases the risk of relapse by 6.36%

Cox regression - MTV no target

Variables in the equation

	B	SE	Wald	Gl	Sig..	Exp(B)	95.0% CI for Exp(B)	
							Inferior	Superior
MTV No target	,000	,001	,032	1	,859	1,000	,999	1,001

Covariate means

	Mean
MTV No target	167,142

p=0.859 does not affect DFS

Variables in the equation

	B	SE	Wald	Gl	Sig.	Exp(B)	95.0% CI for Exp(B)	
							Inferior	Superior
Total MTV	,001	,000	7,903	1	,005	1,001	1,000	1,001

Cox Regression - Total MTV

Covariate means

	Mean
Total MTV	853,970

p=0.005 total MTV is associated with DFS.
For each unit increase in total MTV increases the risk of relapse by 0.0 7%
For every 10 units of increase in total MTV increases the risk of relapse u n 0.71%
For every 100 units of increase in total MTV increases the risk of relapse by 7.29%

ASSOCIATION OF NUCLEAR PARAMETERS WITH OS

Cox Regression - SUVmax

Variables in the equation

	B	SE	Wald	Gl	Sig.	Exp(B)	95.0% CI for Exp(B)	
							Inferior	Superior
SUVmax	-,015	,031	,240	1	,624	,985	,927	1,046

Covariate means

	Mean
SUVmax	12,561

p=0.624 does not affect OS

Cox Regression - SUVpeak

Variables in the equation

	B	SE	Wald	Gl	Sig.	Exp(B)	95.0% CI for Exp(B)	
							Inferior	Superior
SUVpeak	-,054	,064	,703	1	,402	,948	,836	1,074

Covariate means

	Mean
SUVpeak	9,135

p=0.402 does not affect OS

Cox regression - TLG

Variables in the equation

	B	SE	Wald	Gl	Sig.	Exp(B)	95.0% CI for Exp(B)	
							Inferior	Superior
TLG	,000	,000	,713	1	,398	1,000	1,000	1,000

Covariate means

	Mean
TLG	2632,756

p=0.398 does not affect OS

Cox regression - TLG target

Variables in the equation

	B	SE	Wald	Gl	Sig.	Exp(B)	95.0% CI for Exp(B)	
							Inferior	Superior
TLG target	,000	,000	,245	1	,621	1,000	1,000	1,000

Covariate means

	Mean
TLG target	2109,922

p=0.621 does not affect OS

Cox regression - TLG target

Variables in the equation

	B	SE	Wald	Gl	Sig.	Exp(B)	95.0% CI for Exp(B)	
							Inferior	Superior
TLG no target	,000	,000	,603	1	,438	1,000	,999	1,000

Covariate means

	Mean
TLG no target	521,027

p=0.438 does not affect OS

Cox regression - MTV target

Variables in the equation

	B	SE	Wald	Gl	Sig.	Exp(B)	95.0% CI for Exp(B)	
							Inferior	Superior
MTV Target	,000	,000	,086	1	,770	1,000	,999	1,001

Covariate means

	Mean
MTV Target	670,064

p=1,000 does not affect OS

Cox regression - MTV no target

Variables in the equation

	B	SE	Wald	Gl	Sig.	Exp(B)	95.0% CI for Exp(B)	
							Inferior	Superior
MTV No target	,000	,001	,014	1	,907	1,000	,998	1,002

Covariate means

	Mean
MTV No target	175,689

p=1,000 does not affect OS

Cox Regression - Total MTV

Variables in the equation

	B	SE	Wald	Gl	Sig.	Exp(B)	95.0% CI for Exp(B)	
							Inferior	Superior
Total MTV	,000	,000	,394	1	,530	1,000	1,000	1,001

Covariate means

	Mean
Total MTV	874,786

p=1,000 does not affect OS

FOR PATIENTS WHO RELAPSE: MEDIAN FROM TIME TO RELAPSE

Statistical

DFS

N		Percentiles		
Valid	Lost	25	50	75
33	0	321,00	448,00	546,50

FOR PATIENTS WHO DIE: MEDIAN TIME TO EXITUS

Statistical

OS

N		Percentiles		
Valid	Lost	25	50	75
17	1	383,500	789,000	1178,000

ROC CURVES TO SEE IF WE CAN GET CUT-OFF POINTS FOR TLG, TLG TARGET, MTV TARGET, AND TOTAL MTV FROM THE RELAPSED VARIABLE

COR curve

Case Processing Summary

Relapse	Valid N (per list)
Positive[to]	33
Negative	14
Lost	6

Larger values of the test result variables indicate a higher test for a positive actual state.

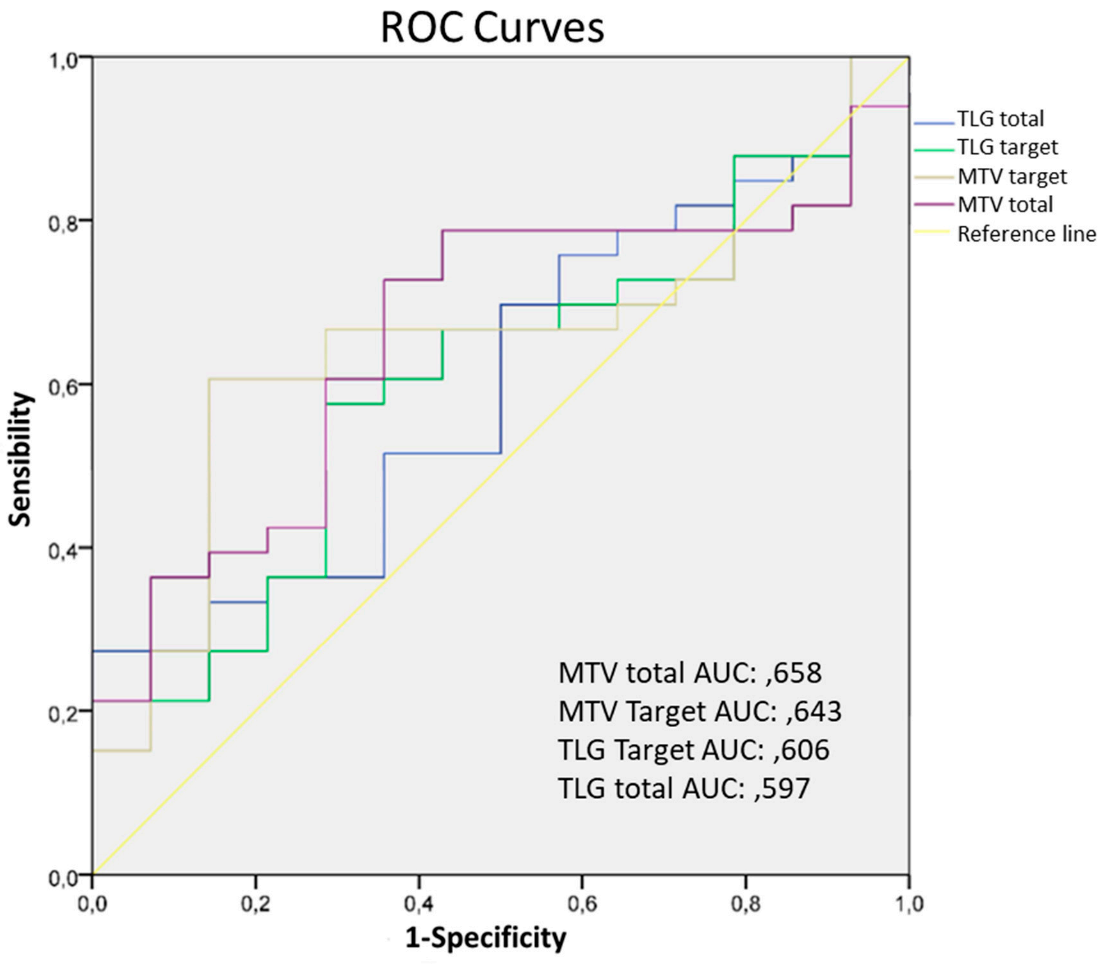

AUC

TLG	,597
TLG target	,606
MTV target	,643
MTV total	,658

Curve coordinates

Test result variables	Positive if it is greater than or equal[to a]	Sensitivity	1 - Specificity
TLG	151,5400	1,000	1,000
	198,9100	,970	1,000
	283,5300	,939	1,000
	345,2800	,939	,929
	381,0550	,909	,929
	462,4450	,879	,929
	554,8600	,879	,857
	592,1400	,848	,857
	623,6800	,848	,786
	771,7850	,818	,786
	923,9450	,818	,714
	1015,2400	,788	,714
	1188,7350	,788	,643
	1319,0900	,758	,643
	1355,7500	,758	,571
	1434,9200	,727	,571
	1516,9250	,697	,571
	1530,1700	,697	,500
	1530,7300	,667	,500
	1593,0600	,636	,500
	1676,1150	,606	,500
	1702,2800	,576	,500
	1714,4550	,545	,500
	1769,9100	,515	,500
	1880,7900	,515	,429
	1943,6050	,515	,357
	2005,6100	,485	,357
	2109,9550	,455	,357
	2208,4900	,424	,357
	2320,4050	,394	,357
	2580,5850	,364	,357
	2888,1900	,364	,286
	2998,5000	,364	,214
	3105,1350	,333	,214
	3287,1100	,333	,143
	3573,3300	,303	,143
	3853,4350	,273	,143
	4028,1150	,273	,071
	4407,3900	,273	,000
	4712,3300	,242	,000
	5250,4450	,212	,000
	5851,4700	,182	,000
	6056,5450	,152	,000
	6187,0250	,121	,000
	6291,3700	,091	,000
	6853,2250	,061	,000
	7540,5750	,030	,000

Curve coordinates

Test result variables	Positive if it is greater than or equal to a	Sensitivity	1 - Specificity
	7758,7600	,000	,000
TLG target	28,7400	1,000	1,000
	29,9750	1,000	,929
	45,4800	,970	,929
	71,7050	,939	,929
	90,7400	,909	,929
	121,4900	,879	,929
	151,9800	,879	,857
	189,6850	,879	,786
	227,8750	,848	,786
	251,4250	,818	,786
	271,2300	,788	,786
	339,4650	,758	,786
	430,8750	,727	,786
	488,2850	,727	,714
	530,2850	,727	,643
	649,8800	,697	,643
	815,9300	,697	,571
	877,7050	,667	,571
	884,8150	,667	,500
	1086,5050	,667	,429
	1290,7700	,636	,429
	1299,5750	,606	,429
	1357,3350	,606	,357
	1413,5050	,576	,357
	1416,8300	,576	,286
	1436,0800	,545	,286
	1470,1750	,515	,286
	1519,9150	,485	,286
	1586,5900	,455	,286
	1715,8950	,424	,286
	1826,2450	,394	,286
	1890,5750	,364	,286
	2150,3800	,364	,214
	2441,0750	,333	,214
	2535,0800	,303	,214
	2737,7200	,273	,214
	3332,3200	,273	,143
	3767,1050	,242	,143
	3813,8800	,212	,143
	3926,0850	,212	,071
	4363,4400	,212	,000
	5206,9650	,182	,000
	5898,5300	,152	,000
	6137,0250	,121	,000
	6279,9250	,091	,000
	6822,8350	,061	,000

Curve coordinates

Test result variables	Positive if it is greater than or equal[to a]	Sensitivity	1 - Specificity
MTV Target	7480,9850	,030	,000
	7693,1800	,000	,000
	1,0100	1,000	1,000
	4,3450	1,000	,929
	7,1650	,970	,929
	9,6300	,939	,929
	14,1700	,909	,929
	17,0550	,879	,929
	22,6000	,848	,929
	28,1750	,818	,929
	32,7950	,818	,857
	39,8650	,788	,857
	62,8050	,788	,786
	101,9700	,758	,786
	132,0000	,727	,786
	147,5000	,727	,714
	170,5000	,697	,714
	198,5000	,697	,643
	230,7750	,667	,643
	269,7750	,667	,571
	292,0000	,667	,500
	317,5000	,667	,429
	352,0000	,667	,357
	396,5000	,667	,286
	428,5000	,636	,286
	432,5000	,606	,286
	441,5000	,606	,214
	462,5000	,606	,143
	476,5000	,576	,143
	509,0000	,545	,143
	545,0000	,515	,143
	558,0000	,485	,143
	589,0000	,455	,143
	628,0000	,424	,143
	653,5000	,394	,143
	667,0000	,364	,143
	712,5000	,333	,143
	898,5000	,303	,143
	1078,0000	,273	,143
	1149,0000	,273	,071
	1203,5000	,242	,071
	1257,0000	,212	,071
	1317,0000	,182	,071
	1426,5000	,152	,071
	1635,0000	,152	,000
	1854,5000	,121	,000
	2151,5000	,091	,000

Curve coordinates

Test result variables	Positive if it is greater than or equal[to a]	Sensitivity	1 - Specificity
Total MTV	2437,5000	,061	,000
	2819,0000	,030	,000
	3116,0000	,000	,000
	66,8200	1,000	1,000
	75,5470	,970	1,000
	85,4765	,939	1,000
	88,2245	,939	,929
	102,7900	,909	,929
	123,5100	,879	,929
	162,5100	,848	,929
	198,1500	,818	,929
	213,2250	,818	,857
	240,0750	,788	,857
	280,2520	,788	,786
	328,5770	,788	,714
	354,3750	,788	,643
	362,7650	,788	,571
	398,0765	,788	,500
	427,8115	,788	,429
	435,7115	,758	,429
	446,8565	,727	,429
	464,0795	,727	,357
	498,1790	,697	,357
	521,9145	,667	,357
	535,6550	,636	,357
	559,9430	,606	,357
	579,4075	,606	,286
	590,7445	,576	,286
	610,3995	,545	,286
	644,7395	,515	,286
	690,1410	,485	,286
	716,9360	,455	,286
	743,3850	,424	,286
	790,9500	,424	,214
	913,8715	,394	,214
	1030,8665	,394	,143
	1099,6300	,364	,143
	1157,9420	,364	,071
	1184,4670	,333	,071
	1223,0210	,303	,071
	1263,0180	,273	,071
	1351,4560	,242	,071
	1480,2585	,212	,071
	1651,3695	,212	,000
	1764,8365	,182	,000
	1864,6155	,152	,000
	2168,9790	,121	,000

Curve coordinates

Test result variables	Positive if it is greater than or equal[to a]	Sensitivity	1 - Specificity
	2454,3650	,091	,000
	2634,2750	,061	,000
	2936,0440	,030	,000
	3135,5880	,000	,000

a. The smallest cut-off value is the minimum observed test value minus 1 and the largest cut-off value is the observed maximum test value plus 1. All other cut-off values are the averages of the two consecutive requested observed test values

DFS WITH MEDIAN CUTOFF

Kaplan-Meier - TLG

Case Processing Summary

TLG_median	Total N	Number of events	Censored	
			N	Percentage
Values less than or equal to the median	25	16	9	36,0%
Values above the median	22	17	5	22,7%
Global	47	33	14	29,8%

Means and medians for survival time

TLG_median	Mean				Median			
			95% confidence interval				95% confidence interval	
	Estimate	Standard error	Lower limit	Upper limit	Estimate	Standard error	Lower limit	Upper limit
Values less than or equal to the median	745,982	93,825	562,085	929,878	594,000	61,682	473,103	714,897
Values above the median	602,958	104,632	397,878	808,307	458,000	28,779	401,593	514,407
Global	701,641	79,364	546,089	857,194	499,000	39,542	421,497	576,503

a. The estimate is limited to the longest survival time, if censored.

Overall comparisons

	Chi-square	Gl	Sig..
Log Rank (Mantel-Cox)	1,003	1	,317

Equal survival distribution test for different levels of TLG_median.

Kaplan-Meier - TLG target

Case Processing Summary

TLGtarget_median	Total N	Number of events	Censored	
			N	Percentage
Values less than or equal to the median	25	15	10	40,0%
Values above the median	22	18	4	18,2%
Global	47	33	14	29,8%

Means and medians for survival time

TLGtarget_median	Mean				Median			
			95% confidence interval				95% confidence interval	
	Estimate	Standard error	Lower limit	Upper limit	Estimate	Standard error	Lower limit	Upper limit
Values less than or equal to the median	804,217	115,981	576,895	1031,540	532,000	45,872	442,091	621,909
Values above the median	570,091	79,335	414,595	725,587	455,000	36,351	383,753	526,247
Global	701,641	79,364	546,089	857,194	499,000	39,542	421,497	576,503

a. The estimate is limited to the longest survival time, if censored.

Overall comparisons

	Chi-square	Gl	Sig.
Log Rank (Mantel-Cox)	1,252	1	,263

Equal survival distribution test for different levels of TLGtarget_median.

Kaplan-Meier - VMT target

Case Processing Summary

MTVtarget_median	Total N	Number of events	Censored	
			N	Percentage
Values less than or equal to the median	26	14	12	46,6%
Values above the median	21	19	2	9,5%
Global	47	33	14	29,8%

Means and medians for survival time

MTVtarget_median	Mean				Median			
	Estimate	Standard error	95% confidence interval		Estimate	Standard error	95% confidence interval	
			Lower limit	Upper limit			Lower limit	Upper limit
Values less than or equal to the median	899,950	121,428	661,952	1137,949	657,000	101,789	457,493	856,507
Values above the median	470,000	41,161	389,325	550,675	455,000	74,001	309,958	600,042
Global	701,641	79,364	546,089	857,194	499,000	39,542	421,497	576,503

a. The estimate is limited to the longest survival time, if censored.

Overall comparisons

	Chi-square	Gl	Sig.
Log Rank (Mantel-Cox)	6,068	1	,014

Equal survival distribution test for different levels of MTV target_median.

Cox regression - MTV target_median

Variables in the equation

	B	SE	Wald	Gl	Sig.	Exp(B)	95.0% CI for Exp(B)	
							Inferior	Superior
MTVtarget_median	,866	,363	5,700	1	,017	2,378	1,168	4,842

Covariate means

	Mean
MTVtarget_median	,457

p=0.017

Presenting values above the median in the MTV target variable increases the risk of relapse by 2.38 times compared to taking values below or equal to the median.

Expressing it as risk reduction:

Cox regression - MTVtarget_median

Variables in the equation

	B	SE	Wald	Gl	Sig.	Exp(B)	95.0% CI for Exp(B)	
							Inferior	Superior
MTVtarget_median	-,866	,363	5,700	1	,017	,421	,207	,856

Covariate means

	Mean
MTVtarget_median	,543

p=0.017

Presenting values less than or equal to the median in the variable MTV targ et reduces by 57.9% (1-Exp(B)=1-0.421=0.579) the risk of relapse compared to taking values above the median.

Kaplan-Meier - Total MTV

Case Processing Summary

MTVtotal_median	Total N	Number of events	Censored	
			N	Percentage

Values less than or equal to the median	26	16	10	38,5%
Values above the median	21	17	4	19,0%
Global	47	33	14	29,8%

Means and medians for survival time

MTVtotal_median	Mean				Median			
	Estimate	Standard error	95% confidence interval		Estimate	Standard error	95% confidence interval	
			Lower limit	Upper limit			Lower limit	Upper limit
Values less than or equal to the median	756,466	93,423	573,358	939,575	616,000	90,802	438,027	793,973
Values above the median	598,750	101,999	398,832	798,668	431,000	30,187	371,834	490,166
Global	701,641	79,364	546,089	857,194	499,000	39,542	421,497	576,503

a. The estimate is limited to the longest survival time, if censored.

Overall comparisons

	Chi-square	Gl	Sig.
Log Rank (Mantel-Cox)	3,240	1	,072

Equal survival distribution test for different levels of MTVtotal_median.
