# Supplementary material for: Prognostic Value of Pretreatment 18F-FDG-PET/CT Metabolic Parameters in Advanced High-Grade Serous Ovarian Cancer
Source: Cancers (Basel). 2025 Feb 19;17(4):698. doi: 10.3390/cancers17040698 (PMC11853401; doi:10.3390/cancers17040698)
Supplement: Supplementary file 1 [file cancers-17-00698-s001.zip › File S5. Results 2.pdf]

PROGRESSION-FREE SURVIVAL ACCORDING TO VMTtotal_roc

Point 427.8115 has been considered as cut-off, this point has a sensitivity of 78.8% (classifies well 78.8% of patients who relapse) and a specificity of 57.1% (classifies well 57.1% ofthose who do not relapse).

Kaplan-Meier - MTVtotal_roc

Case Processing Summary

MTVtotal_roc	Total N	Number of	Censored	
			N	Percentage
values less than or equal to the cut-off point	15	7	8	53.3%
Values greater than the code point	32	26	6	18.8%
Global	47	33	14	29,8%

Table of survival

MTVtotal_roc		Hour	State	Cumulative proportion that survives over time: Estimackin	Standard error	N of cumulative events	N of remaining
Values less than or equal to cut-off point	1	261,000	Yes	,933	,064	1	14
	2	277,000	No	.	.	1	13
	3	292,000	Yes	,862	,091	2	12
	4	367,000	Yes	,790	,108	3	11
	5	432,000	No	.	.	3	10
	6	448,000	Yes	,711	,123	4	9
	7	605,000	No	.	.	4	8
	8	631,000	No	.	.	4	7
	9	657,000	Yes	,609	,141	5	6
	10	680,000	Yes	,508	,150	6	5
	11	736,000	No	.	.	6	4
	12	770,000	No	.	.	6	3
	13	954,000	No	.	.	6	2
	14	1347,000	Yes	,254	,194	7	1
	15	1393,000	No	.	.	7	0
Values higher than point cutting	1	74,000	No	.	.	0	31
	2	133,000	Yes	,968	,032	1	30
	3	291,000	Yes	,935	,044	2	29
	4	313,000	Yes	,903	,053	3	28
	5	314,000	Yes	,871	,060	4	27
	6	315,000	Yes	,839	,066	5	26
	7	318,000	No	.	.	5	25
	8	319,000	Yes	,805	,071	6	24
	9	323,000	Yes	,772	,076	7	23
	10	347,000	Yes	,738	,080	8	22
	11	361,000	Yes	,705	,083	9	21
	12	381,000	Yes	,671	,085	10	20
	13	404,000	Yes	,637	,087	11	19
	14	428,000	Yes	,604	,089	12	18
	15	431,000	Yes	,570	,090	13	17
	16	455,000	Yes	,537	,091	14	16
	17	458,000	Yes	,503	,091	15	15
	18	469,000	Yes	,470	,091	16	14
	19	490,000	Yes	,436	,091	17	13
	20	499,000	Yes	.	.	18	12
	21	499,000	Yes	,369	,088	19	11
	22	530,000	Yes	,335	,086	20	10
	23	532,000	Yes	,302	,084	21	9
	24	561,000	Yes	,268	,081	22	8
	25	594,000	Yes	,235	,078	23	7
	26	616,000	Yes	,201	,073	24	6
	27	673,000	Yes	,168	,068	25	5
	28	693,000	No	.	.	25	4
	29	712,000	No	.	.	25	3
	30	715,000	No	.	.	25	2
	31	803,000	Yes	,084	,068	26	1
	32	1650,000	No	.	.	26	0

Means and medians for survival time

	Media'	Median

			95% confidence interval				95% confidence interval	
	Estimation	Standard	Lower limit	Upper limit	Estimation	Standard	Lower limit	Upper limit
values less than or equal to the code point	931,389	132,439	671,809	1190,969	1347,000	377,608	606,889	2087,111
Values above the torte point	564,148	76,596	414,019	714,277,85 7,194	469,000	40,059	390,485	547,515
Global	701,641	79,364	546,089		499,000	39,542	421,497	576,503

to. The estimate is limited to the longest survival time, if censored.

Overall comparisons

	Chi-square	Gl	Sig.
Log Rank (Mantel-Cox)	5,259	1	,022

Equal survival distribution test for different levels of MTVtotal_roc.

p=0 . 022 there are differences in survival according to MTVtotal roc

Regression by Cox - MTVtotal_roc

Variables in the equation

	B	SE	Wald	Gl	Sig.	Exp(B)	95.0% CI for Exp(B)	
							Inferior	Superior
MTVtotal_roc	-,957	,433	4,897	1	,027	,384	,164	,896

Covariate means

	Mean
MTVtotal_roc	,326

p=0.027 presenting values less than or equal to the cut-off point reduces the risk of relapse by 61.6% (1-Exp(B)=1-0.384) compared to taking values higher than the cut-off point.
